# Functional Roles of the Non-Catalytic Calcium-Binding Sites in the N-Terminal Domain of Human Peptidylarginine Deiminase 4

**DOI:** 10.1371/journal.pone.0051660

**Published:** 2013-01-30

**Authors:** Yi-Liang Liu, I-Chen Tsai, Chia-Wei Chang, Ya-Fan Liao, Guang-Yaw Liu, Hui-Chih Hung

**Affiliations:** 1 Department of Life Sciences and Institute of Genomics and Bioinformatics, National Chung-Hsing University, Taichung, Taiwan; 2 Graduate Institute of Biochemical Sciences and Biotechnology, Chaoyang University of Technology Taichung, Taichung, Taiwan; 3 Institute of Microbiology & Immunology, Chung Shan Medical University, and Division of Allergy, Immunology, and Rheumatology, Chung Shan Medical University Hospital, Taichung, Taiwan; 4 Agricultural Biotechnology Center (ABC), National Chung Hsing University, Taichung, Taiwan; Concordia University Wisconsin, United States of America

## Abstract

This study investigated the functional roles of the N-terminal Ca^2+^ ion-binding sites, in terms of enzyme catalysis and stability, of peptidylarginine deiminase 4 (PAD4). Amino acid residues located in the N-terminal Ca^2+^-binding site of PAD4 were mutated to disrupt the binding of Ca^2+^ ions. Kinetic data suggest that Asp155, Asp157 and Asp179, which directly coordinate Ca3 and Ca4, are essential for catalysis in PAD4. For D155A, D157A and D179A, the *k*_cat_/*K*_m,BAEE_ values were 0.02, 0.63 and 0.01 s^−1^mM^−1^ (20.8 s^−1^mM^−1^ for WT), respectively. Asn153 and Asp176 are directly coordinated with Ca3 and indirectly coordinated with Ca5 via a water molecule. However, N153A displayed low enzymatic activity with a *k*_cat_ value of 0.3 s^−1^ (13.3 s^−1^ for wild-type), whereas D176A retained some catalytic power with a *k*_cat_ of 9.7 s^−1^. Asp168 is the direct ligand for Ca5, and Ca5 coordination by Glu252 is mediated by two water molecules. However, mutation of these two residues to Ala did not cause a reduction in the *k*_cat_/*K*_m,BAEE_ values, which indicates that the binding of Ca5 may not be required for PAD4 enzymatic activity. The possible conformational changes of these PAD4 mutants were examined. Thermal stability analysis of the PAD4 mutants in the absence or presence of Ca^2+^ indicated that the conformational stability of the enzyme is highly dependent on Ca^2+^ ions. In addition, the results of urea-induced denaturation for the N153, D155, D157 and D179 series mutants further suggest that the binding of Ca^2+^ ions in the N-terminal Ca^2+^-binding site stabilizes the overall conformational stability of PAD4. Therefore, our data strongly suggest that the N-terminal Ca^2+^ ions play critical roles in the full activation of the PAD4 enzyme.

## Introduction

Peptidylarginine deiminase (PAD; protein-arginine deiminase, EC 3.5.3.15) is a Ca^2+^-dependent enzyme that catalyzes the conversion of arginyl to citrullyl residues in proteins, which is accompanied by the production of ammonia. The deiminase family is involved in a post-translational process called citrullination, which has significant effects on the physiological and functional properties of target proteins and plays a regulatory role in cell differentiation and development ([1]–[3], and references there in).

PAD plays essential roles in cell differentiation [4], nerve growth [5], [6], embryonic development [7], cell apoptosis [8]–[10] and gene regulation [11], [12]. PAD has broad substrate specificity; PAD and citrullinated proteins are associated with human diseases, such as psoriasis, multiple sclerosis, rheumatoid arthritis (RA) and Alzheimer's disease. In addition, various types of cancers are also associated with PAD enzymes and their citrullinated targets [13]–[17]. Five isoforms of PAD (PAD1-4 and PAD6) have been characterized, and they have different tissue distributions [3], [4], [18]–[23]. PAD1 is found in the skin epidermis and citrullinates keratins and filaggrins [4], [18], [24]. PAD2 is found in the brain, nervous system and muscle tissues [19]. PAD3 is localized in hair follicles, where trichohyalin citrullination occurs during hair follicle hardening [20], [25]. PAD4 is found in granulocytes; monocytes; macrophages; citrullinated histones H2A, H3 and H4; and nucleophosmin/B23 [11], [12], [21], [26]. Finally, PAD6 has been identified in embryonic stem cells and oocytes [23].

Studies of protein citrullination and PAD enzymes have attracted increased attention during the last ten years. Two major issues are of concern. The first issue is that high PAD4 activity and citrullinated proteins are associated with the pathogenesis of rheumatoid arthritis (RA), which is an autoimmune disease [27]. An overabundance of autoantibodies against citrullinated proteins is often detected in the blood of RA patients [28]. In Japan, a case control study demonstrated that the *PAD4* haplotype associated with susceptibility to RA increases production of deaminated peptides acting as autoantigens [27], [29]. Notably, PAD4 is autocitrullinated *in vitro* and *in vivo*, and this modification inactivates enzyme function and augments its recognition by human autoantibodies [30].

The second issue is that PAD4 is involved in histone demethylimination (citrullination) and induces changes in chromatin structure, which affects the transcriptional regulation of genes. Histones are often methylated, and PAD4 removes the methyl group from modified arginine residues of histones and causes a decrease in gene expression. As PAD4 may play an antagonistic role in histone methylation, it is considered to be a transcriptional co-repressor [31]. PAD4 is involved in the repression of p53 target gene expression, which interacts with the C-terminus of p53 and regulates p53 target genes [32], [33]. Because PAD4 has histone methylarginine deiminase activity, PAD4 activity causes negative regulation of downstream p53 target genes, including the p21 protein.

**Figure 1:**
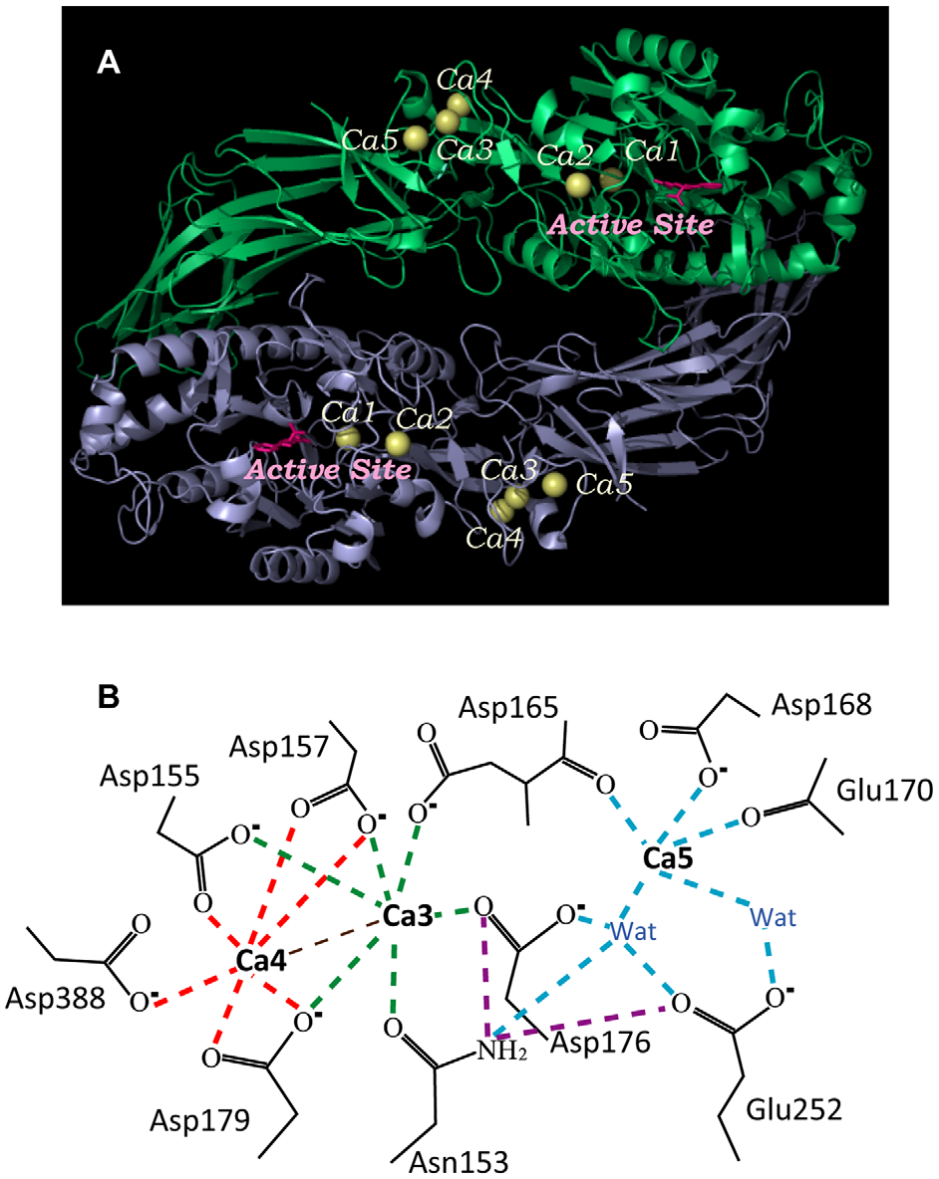
Crystal structure and N-terminal Ca^2+^-binding site in the human PAD4 enzyme. **A.** A homodimer of Ca^2+^-bound human PAD4 with synthetic substrates (PDB code 1WDA). Five calcium ions are colored yellow; the substrate is represented as a ball-and-stick model and colored pink. The active site consists of Ca1, Ca2 and substrate complex. **B.** N-terminal Ca^2+^-binding sites of PAD4. The binding network of these ligands is represented by a dashed line. Ligand interactions for Ca3, Ca4, and Ca5 are green, red, and cyan, respectively. The hydrogen bonds between Asn153 and Asp176 are shown in purple. This figure was generated using PyMOL (DeLano Scientific LLC; San Carlos, CA).

PAD4 is a homodimeric enzyme with a monomeric molecular weight of 74 kDa. X-ray data demonstrate that five Ca^2+^ ions, designated Ca1, Ca2, Ca3, Ca4 and Ca5, reside in the protein (Figure 1A) [34]. Ca1 and Ca2 are located at the bottom of the active cleft in the C-terminal domain. The amino acid residues for Ca1 and Ca2 binding, except for PAD6, are largely conserved in all PAD enzymes. PAD4 has an absolute requirement of Ca^2+^ ions for enzyme activity. The conformational changes that occur around the active site strongly suggest that the binding of Ca1 and Ca2 to the acidic concave surface of the C-terminal domain is crucial for recognition of the substrate [34]. The enzyme uses an active site Cys to initiate the chemical reaction with nucleophilic attack by the thiol group of Cys645 on the Cδ atom of the peptidylarginine [35]–[37]. In addition to the active-site ions (Ca1 and Ca2), three Ca^2+^ ions, Ca3, Ca4 and Ca5, reside on the molecular surface of subdomain 2 in the N-terminal domain. Ca3 and Ca4 interact with each other and are coordinated by Asn153, Asp155, Asp157, Asp165, Asp176 and Asp179, and Asp155, Asp157, Asp179 and Asp388, respectively. Ca5 is coordinated by Asp168, Glu170, Asp252 and two water molecules (Figure 1B). Because this region mainly consists of acidic residues, the binding of Ca3, Ca4 and Ca5 in the area may stabilize the structure. However, the exact role of these Ca^2+^ ions in enzyme catalysis and activation is still unknown.

Our recent studies demonstrate the functional role of dimerization in the regulation of PAD4 activity. We propose that dimerization of PAD4 is essential for full enzymatic activity and calcium binding cooperativity [38]. In this paper, we aimed to explore the functional roles of the N-terminal Ca^2+^ ions. To address these questions, we created a series of PAD4 mutants of residues Asn153, Asp155, Asp157, Asp165, Asp168, Asp176, Asp179, Glu252 and Asp388, which are located in the Ca3, Ca4 and Ca5 binding network (Figure 1B). The ligand-Ca^2+^ interactions at these Ca^2+^ binding sites were disrupted by site-directed mutagenesis, which resulted in conformational changes and a loss of enzyme activity. Our data strongly suggest that the N-terminal Ca^2+^ ions are essential for full activation of the enzyme.

## Results

### Kinetic properties of human PAD4 wild-type and alanine mutants at the Ca3, Ca4 and Ca5 binding sites

The N-terminal Ca^2+^-binding residues are Asn153, Asp155, Asp157, Asp165, Asp168, Asp176, Asp179, Glu252 and Asp388 (Figure 1B). The following PAD4 single mutants were constructed: N153A, D155A, D157A, D165A, D168A, D176A, D179A, E252A and D388A.

The kinetic parameters of human wild-type (WT) PAD4 and alanine mutants are presented in Table 1. The Michaelis constant of the *in vitro* substrate BAEE (*K*_m,BAEE_), catalytic constant (*k*_cat_) and specificity constant (*k*_cat_/*K*_m,BAEE_) of the WT enzyme were 0.64 mM, 13.3 s^−1^ and 20.8 s^−1^mM^−1^, respectively. However, for most PAD4 alanine mutants, the *K*_m,BAEE_ values were significantly elevated with decreased *k*_cat_ and *k*_cat_/*K*_m,BAEE_ values, which indicates that the catalytic efficiency of the enzyme was significantly reduced by these mutations.

**Table 1:** Kinetic parameters of PAD4 wild-type and alanine-mutant enzymes at the Ca3, Ca4 and Ca5 binding sites.

*Ca ligand*	PAD4	K_m,BAEE_ a (mM)	k_cat_ a (s^–1^)	k_cat_/K_m,BAEE _(s^–1^mM^–1^)	K_0.5,Ca_ (mM)	*h* coefficient
	**WT**	0.64±0.09	13.3±0.3	20.8	0.61±0.02	2.7±0.2
*Ca3-Water*	**N153A**	4.61±1.16	0.3±0.01	0.07	7.69±0.35	1.8±0.2
*Ca3-Ca4*	**D155A**	4.73±0.87	0.1±0.03	0.02	7.75±1.68	1.0±0.2
*Ca3-Ca4*	**D157A**	5.84±1.08	3.7±0.1	0.63	6.71±0.12	2.6±0.1
*Ca3*	**D165A**	7.72±0.72	9.2±0.3	1.19	4.49±0.23	2.4±0.3
*Ca5*	**D168A**	2.73±0.30	13.0±1.0	4.76	2.58±0.12	2.7±0.2
*Ca3-Water*	**D176A**	4.07±0.52	9.7±0.6	2.38	5.20±0.25	2.3±0.2
*Ca3-Ca4*	**D179A**	9.89±0.79	0.1±0.01	0.01	6.89±0.66	1.3±0.1
*Water-Ca5*	**E252A**	1.29±0.24	12.7±1.3	9.84	3.60±0.55	2.4±0.1
*Ca4*	**D388A**	3.72±0.33	8.2±0.3	2.20	4.35±0.33	2.2±0.3
*Ca5*	**D168A/D252A**	1.58±0.14	10.2±0.3	6.46	2.24±0.10	2.5±0.1

a kinetic analysis of *K*_m,BAEE_ and *k*_cat_ was determined in the presence of 10 mM Ca^2+^.

In the N-terminal Ca^2+^-binding site, the carboxylic side-chain of Asp155, Asp157 and Asp179 are directly coordinated by Ca3 and Ca4 (Figure 1B). Mutation of these amino acid residues to alanine resulted in reduced enzymatic activity. For D155A and D179A, the *K*_m,BAEE_ value increased 7- and 15-fold, respectively; the *k*_cat_ value was reduced to 0.1 s^−1^, which is only 1% of the WT enzyme activity; and the *k*_cat_/*K*_m,BAEE_ values were 0.02 and 0.01 s^−1^mM^−1^ (20.8 s^−1^mM^−1^ for WT), respectively, indicating that D155A and D179A completely lost their catalytic power (Table 1). For D157A, which retained more activity than the other two mutants, the *K*_m,BAEE_ value increased 9-fold and *k*_cat_ value was reduced to 3.7 s^−1^, which is 28% of the WT enzyme. However, the *k*_cat_/*K*_m,BAEE_ value of this mutant enzyme was only 0.63 s^−1^mM^−1^ (20.8 s^−1^mM^−1^ for WT), indicating its poor catalytic efficiency (Table 1). These data strongly suggest that Asp155, Asp157 and Asp179 play a critical role in the catalysis of PAD4. Therefore, mutation of these ligands for Ca3 and Ca4 had a significant impact on catalysis.

Asp165 and Asp388 are the ligands that are coordinated with Ca3 and Ca4, respectively (Figure 1B). A previous study demonstrated that Asp388 is not required for enzyme activity because the D388A mutant displays ∼80% of WT enzymatic activity [34]. In this study, kinetic data revealed that the D165A and D388A mutants retained partial catalytic power with an increase in *K*_m,BAEE_ values of approximately 12- and 6-fold and a slight decrease in *k*_cat_ values, which ranged from 8 to 9 s^−1^. However, the *k*_cat_/*K*_m,BAEE_ values of these two mutant enzymes were only 1.19 and 2.20 s^−1^mM^−1^ (20.8 s^−1^mM^−1^ for WT), respectively, indicating that ablation of the side chain of Asp165 and Asp388 also cause a noteworthy effect on the catalytic efficiency of the enzyme.

Asn153 and Asp176 are directly coordinated with Ca3 and indirectly coordinated with Ca5 via a water molecule (Figure 1B). N153A displayed low catalytic power; the *K*_m,BAEE_ for the mutant increased and *k*_cat_ of the mutant was reduced to 2% of the WT enzyme. The *k*_cat_/*K*_m,BAEE_ value of N153A was only 0.07 s^−1^mM^−1^ (20.8 s^−1^mM^−1^ for WT). Although D176A retained some catalytic power with a *k*_cat_ value of 9.7 s^−1^ (13.3 s^−1^ for WT), the *k*_cat_/*K*_m,BAEE_ value of this mutant was only 2.38 s^−1^mM^−1^ (20.8 s^−1^mM^−1^ for WT), which is 10% of the WT enzyme.

Asp168 is the direct ligand for Ca5, and Ca5 coordination by Glu252 is mediated by two water molecules. However, mutation of these two residues to Ala did not cause a reduction in *k*_cat_ but resulted in a slight elevation in *K*_m,BAEE_ (Table 1). The *k*_cat_/*K*_m,BAEE_ values of these two mutants were 4.36 and 9.84 s^−1^mM^−1^, respectively, indicating that ablation of the side chain of Asp168 and Glu252 did not cause a considerable effect on the catalytic efficiency of the enzyme. The double mutant, D168A/E252A, displayed a slight decrease in *k*_cat_, with kinetic properties similar to that of the respective single mutants (Table 1). These kinetic results of the single and double mutants, with respect to the Ca5 binding site, suggest that the binding of Ca5 may not be required for PAD4 enzyme activity.

### Kinetic properties of conservative PAD4 mutations at the Ca3 and Ca4 binding sites

The N153A, D155A, D157A and D179A enzymes displayed poor catalytic efficiency (Table 1). To examine the effect of the side chains of these residues, a series of conservative mutants, including N153D, N153Q, D155E, D155N, D157E, D157N, D179E and D179N, were created; their kinetic parameters are shown in Table 2. For the N153 series mutants, the *k*_cat_ values of N153D and N153Q significantly recovered to 5.0 and 3.4 s^−1^ (0.3 s^−1^ for N153A), respectively; however, the *K*_m,BAEE_ for both mutants was elevated and similar to N153A. The *k*_cat_/*K*_m,BAEE_ values of these two mutants were only 1.45 and 0.86 s^−1^mM^−1^ (0.07 s^−1^mM^−1^ for N153A), respectively, indicating that the catalytic efficiency of N153A was not significantly recovered by substitution of Ala with Asp or Gln at this position.

**Table 2:** Kinetic parameters of PAD4 conservative mutant enzymes at the Ca3 and Ca4 binding sites.

PAD4	*K*_m,BAEE_ a (mM)	*k*_cat_ a (s^−1^)	*k*_cat_/*K*_m,BAEE_ (s^−1^mM^−1^)	*K*_0.5,Ca_ (mM)	*h* coefficient
**WT**	0.64±0.09	13.3±0.3	20.8	0.61±0.02	2.7±0.2
**N153A**	4.61±1.16	0.3±0.01	0.07	7.69±0.35	1.8±0.2
**N153D**	3.45±0.60	5.0±0.2	1.45	3.84±0.12	2.4±0.1
**N153Q**	3.97±0.48	3.4±0.3	0.86	6.39±0.27	2.3±0.2
**D155A**	4.73±0.87	0.1±0.03	0.02	7.75±1.68	1.0±0.2
**D155E**	4.16±0.50	0.1±0.004	0.02	6.01±0.46	1.8±0.2
**D155N**	8.70±1.72	0.4±0.02	0.05	7.02±0.63	1.8±0.2
**D157A**	5.84±1.08	3.7±0.1	0.63	6.71±0.12	2.6±0.1
**D157E**	0.71±0.11	13.8±0.2	19.4	1.35±0.08	2.4±0.2
**D157N**	4.59±0.42	6.9±0.9	1.50	4.84±0.67	2.7±0.6
**D179A**	9.89±0.79	0.1±0.01	0.01	6.89±0.66	1.3±0.1
**D179E**	0.65±0.06	10.9±0.3	16.8	0.83±0.04	2.1±0.1
**D179N**	2.52±0.63	0.1±0.01	0.04	6.19±1.55	1.0±0.2

a kinetic analysis of *K*_m,BAEE_ and *k*_cat_ was determined in the presence of 10 mM Ca^2+^.

Moreover, the catalytic power of D155A could not be rescued by the replacement of Ala with Glu or Asn. For N155 series mutants, the *K*_m,BAEE_ value of the D155E and D155N mutants increased 7- and 14-fold, respectively, and the *k*_cat_ values ranged from 0.1 to 0.4 s^−1^, which is only 1 to 3% of the WT enzyme. The *k*_cat_/*K*_m,BAEE_ values of these two mutants were merely 0.02 and 0.05 s^−1^mM^−1^ (0.02 s^−1^mM^−1^ for D155A), respectively, which suggests that the coordination of the carboxylic side chain of Asp155 with Ca3 and Ca4 is critical for the correct geometry of the N-terminal Ca^2+^ binding site.

However, for Asp157 and Asp179, these residues could be substituted by Glu but not Asn. The D157N and D179N enzymes displayed a *k*_cat_/*K*_m,BAEE_ value of 1.5 and 0.04 s^−1^mM^−1^ (0.63 s^−1^mM^−1^ for D157A and 0.01 s^−1^mM^−1^ for D179A), respectively. In contrast, the D157E and D179E mutants displayed a kinetically fully functional enzyme relative to WT. The *K*_m,BAEE_ value of D157E and D179E was similar to WT, and the *k*_cat_ values of both mutants significantly recovered to 13.8 and 10.9 s^−1^ (3.7 s^−1^ for D157A and 0.1 s^−1^ for D179A), respectively. The *k*_cat_/*K*_m,BAEE_ values of these two mutants were 19.4 and 16.8 s^−1^mM^−1^ (20.8 s^−1^mM^−1^ for WT), respectively, indicating that the catalytic efficiency of these two mutant enzymes were at the same level as that of WT, which suggests a critical role for the carboxylic side chain at residues 157 and 179.

### Cooperative Effect of Ca^2+^ ions on WT and mutant PAD4

PAD4 cooperatively binds Ca^2+^ ions [38]. The initial velocity of PAD4 measured at various Ca^2+^ concentrations displayed sigmoidal kinetics. Table 1 shows the results acquired from fitting the sigmoidal curves to the Hill equation. The half-saturation constant for Ca^2+^ (*K*_0.5,Ca_) and degree of cooperativity of calcium binding (*h*) were estimated. The *K*_0.5,Ca_ of WT PAD4 was 0.6 mM, and the *h* value was ∼2.7. Similar to *K*_m,BAEE_, the PAD4 alanine mutants had *K*_0.5,Ca_ values greater than WT (Table 1). However, most of the mutants retained Ca^2+^ binding cooperativity with an *h* value >2. For the D165A, D168A, D176A, E252A and D388A mutant enzymes, the mutants conserved most of the catalytic power with remarkable cooperativity. For the N153A, D155A and D179A mutants, which were the least active mutants, cooperativity was significantly reduced. The cooperative Ca^2+^ binding of the D155A and D179A mutants was nearly abolished with an *h* value near 1, which indicated that these mutants have become non-cooperative enzymes. For the D157A enzyme, which was less active than WT, the initial velocities with various Ca^2+^ concentrations still displayed strong sigmoidal kinetics, which suggests that the cooperative effect of Ca^2+^ on the enzyme still occurred with a high *K*_0.5,Ca_ value of 6.7 mM (Table 1).

For the N153 series mutants, the *h* values of N153D and N153Q were >2 with moderate activities compared to WT (up to 25% of the WT *k*_cat_, Table 2). However, the *K*_0.5,Ca_ values for both mutants were greater than WT and similar to N153A, which suggests that cooperativity of the N153D and D153Q enzymes occurred at high calcium concentrations (Table 2). For the N155 series mutants, the *h* values of D155E and D155N were <2 with low *k*_cat_ values; the *K*_0.5,Ca_ values of both were not reduced and similar to that of N155A. However, for Asp157 and Asp179, which could be substituted with Glu at these sites, the *h* values of the D157E and D179E mutants were >2 and had similar catalytic power compared to WT. The *K*_0.5,Ca_ values for both mutants were reduced and similar to that of WT, which suggests that the cooperativity of the D157E and D179E enzymes was maintained at normal calcium ion concentrations (Table 2). The *K*_0.5,Ca_ values of the D157N and D179N mutants were greater than that of WT and similar to those of their respective alanine mutants. The *h* value of D157N was 2.7 and had a *k*_cat_ that was half of WT. The *h* value of D179N was reduced to 1 and had a large *K*_0.5,Ca_ value and little enzyme activity, which indicates that this Asp residue could not be replaced by Asn.

### Quaternary structure of WT and mutant PAD4 enzymes

We have demonstrated that active PAD4 is in a dimeric form not in a monomeric form [38]. Thus, mutant PAD4 enzymes with low enzymatic activity were examined by analytical ultracentrifugation to investigate their possible quaternary structural changes. All of the mutants existed as dimers in solution (Figure S1). N153A predominantly existed as a dimer with a small amount of monomers. The D155A, D157A and D179A enzymes were still dimeric, which indicated that the low enzymatic activity of these mutants was not caused by dissociation of the enzyme dimers.

### Biophysical analysis of WT and mutant PAD4 enzymes

**Figure 2:**
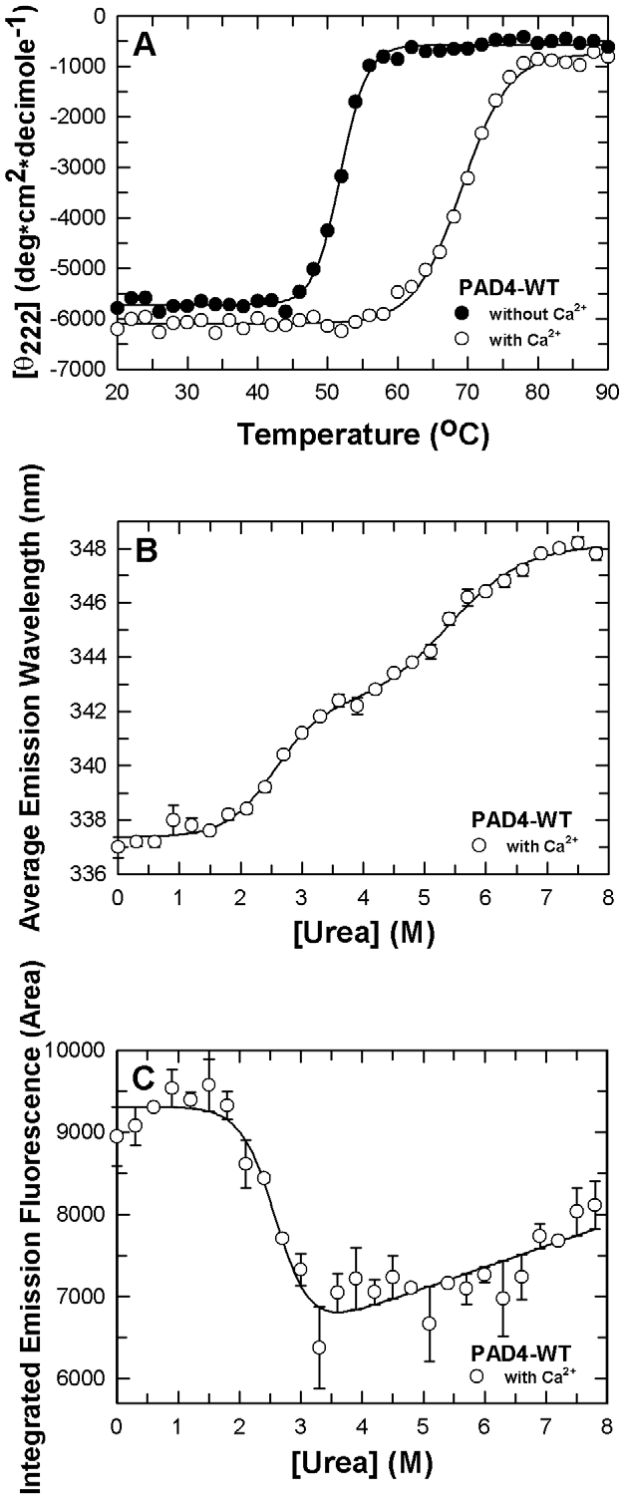
Conformational stability of human PAD4 wild-type enzyme. **A.** Thermal denaturation of WT PAD4 in the presence or absence of 10 mM Ca^2+^ (open and closed circles, respectively). The experimental data are the mean residue ellipticity at 222 nm ((Θ)_222_) monitored by far-UV CD. The urea denaturation of WT PAD4 in the presence of 10 mM Ca^2+^ was monitored by intrinsic protein fluorescence. **B.** Experimental data and curve fitting of average emission wavelength. **C.** Experimental data and curve fitting of integrated emission fluorescence. All data in panels B and C were fitted by either two-state or three-state models. The fitting results and residues are shown as a solid line with error bars.

To examine the possible conformational changes occurring in the PAD4 mutant enzymes, the thermal stability and urea-induced denaturation were examined by far-UV CD and fluorescence, respectively. First, we examined the thermal stability of the PAD4 WT enzyme in the absence and presence of Ca^2+^ (Figure 2A); the *T*_m_ values are presented in Table 3. The thermal stability of WT PAD4 with Ca^2+^ was superior to the enzyme without Ca^2+^. The *T*_m_ of the enzyme with Ca^2+^ was 17°C greater than without Ca^2+^, which indicated that the conformational stability of the enzyme is highly dependent on Ca^2+^ ions. Next, we examined the conformational stability of WT PAD4 by chemical denaturation (Figures 2B and 2C) and determined the thermodynamic parameters (Table 3). In all of the urea-induced denaturation experiments, the WT and mutant PAD4 enzymes were preincubated with 10 mM Ca^2+^ because they readily precipitated if they were denatured by urea under Ca^2+^-free conditions. The urea-induced denaturation of WT PAD4, which is represented by curves of the average fluorescence emission wavelength (Figure 2B) and integrated emission fluorescence (Figure 2C), demonstrated biphasic and monophasic behaviors. The urea concentrations at half-maximal denaturation, [Urea]_0.5_, for the first and second phases ([Urea]_0.5,N→I_ and [Urea]_0.5,I→U_, respectively) of the average fluorescence emission wavelength (Figure 2B) were 2.5 and 5.4 M (Table 3). [Urea]_0.5_ for the monophasic curve of the integrated emission fluorescence ([Urea]_0.5,N→U_) (Figure 2C) was 2.6 M, which coincided with the first phase of the average fluorescence emission wavelength (Figure 2B).

**Table 3:** Thermal stability and thermodynamic parameters of PAD4 wild-type and mutant enzymes during urea-induced denaturation.

	*T*_m_^a^ (°C)			Midpoint of the Urea-Induced Denaturation with 10 mM Ca^2+^		
PAD4	b(−)Ca^2+^	b(+)Ca^2+^	Δ*T*_m_	[Urea]_0.5,N→I_ c (M)	[Urea]_0.5,I→U_ c (M)	[Urea]_0.5,N→U_ d(M)
**WT**	52	69	+17	2.5±0.7	5.4±1.5	2.6±1.0
**N153A**	54	61	+7	1.1±0.9	5.2±6.3	1.6±2.3
**N153Q**	54	60	+6	0.9±0.4	4.9±0.9	1.6±1.0
**D155A**	54	59	+5	1.2±0.6	4.6±1.6	2.0±1.2
**D155E**	53	59	+6	1.2±1.0	4.5±2.2	1.3±0.4
**D157A**	54	60	+6	1.6±1.7	4.3±1.0	2.7±1.2
**D157E**	53	64	+11	2.1±0.5	5.3±1.4	2.2±1.1
**D179A**	54	62	+8	1.6±0.7	5.0±2.0	2.1±4.2
**D179E**	52	64	+12	2.2±0.8	5.1±2.2	2.4±1.6

the data were derived from Figure 3, which were monitored using circular dichroism spectrometry.

b (−), without Ca^2+^; (+) with 10 mM Ca^2+^.

c the data were derived from the fitting results of Figure 2B and left panels of Figures 4 and 5, which were monitored using the averaged emission wavelength of fluorescence spectrometry.

d the data were derived from the fitting results of Figure 2C and right panels of Figures 4 and 5, which were monitored using the integrated area of fluorescence spectrometry.

For the PAD4 mutants, the thermal stability of these enzymes without Ca^2+^ was unchanged (Figure 3); the *T*_m_ values of the mutants were approximately 52–54°C, which is similar to that of WT (Table 3). In presence of Ca^2+^, most of the mutant enzymes were less stable than WT (Figure 3); the *T*_m_ values of these mutants with Ca^2+^ were ∼59–64°C, which is 5–9°C less than WT (Table 3). These data indicate that the thermal stabilities of the mutant enzymes were significantly reduced, and this phenomenon may be because of weaker binding of the Ca^2+^ ions. However, for D157E and D179E, which have kinetic properties that are similar to the level of WT, their thermal stability in the presence of Ca^2+^ was higher than PAD4 mutants that displayed little enzyme activity (Table 2).

**Figure 3:**
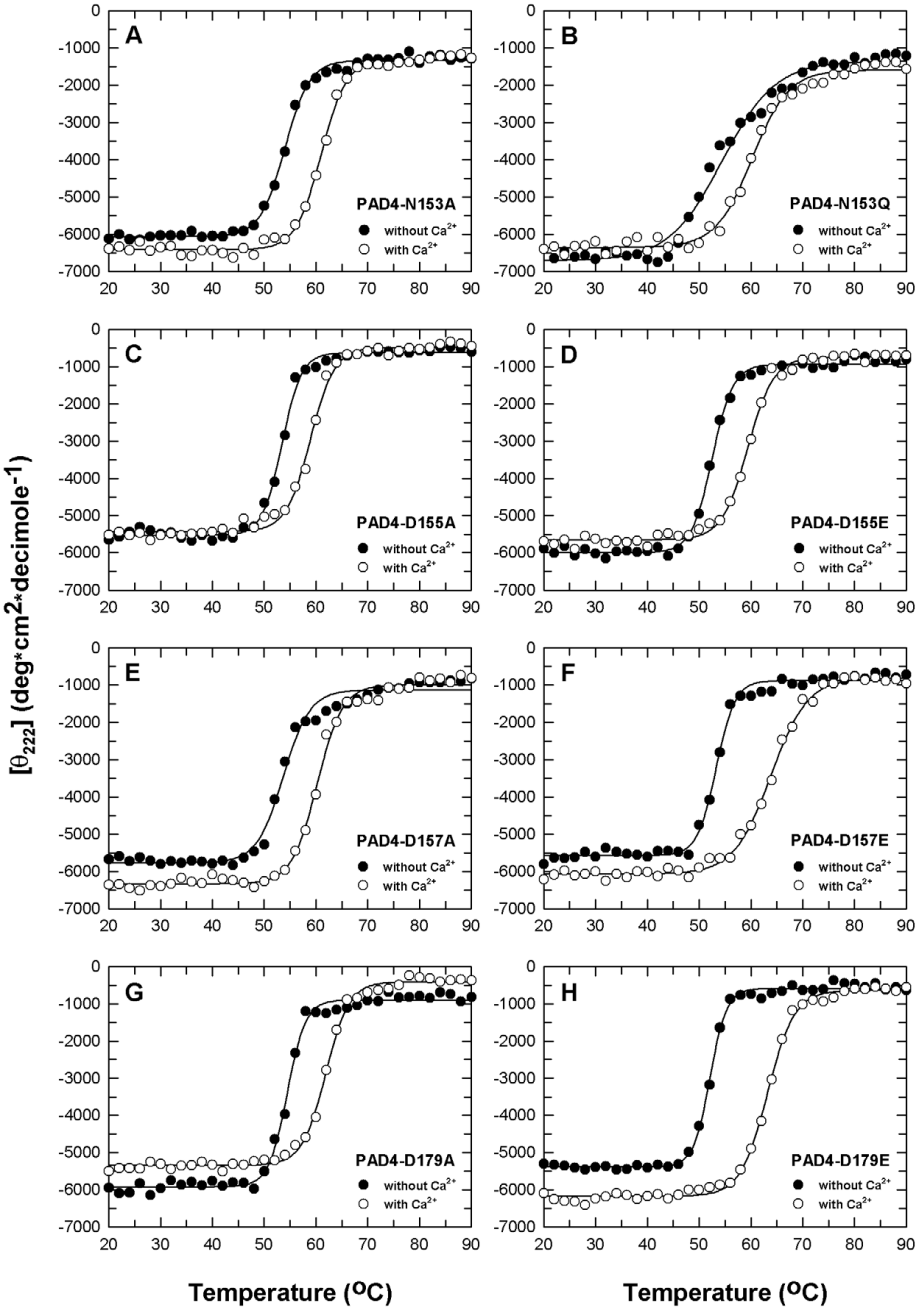
Thermal stability of PAD4 mutant enzymes in the absence or presence of calcium ions. Thermal denaturation of PAD4 mutant enzymes in the presence or absence of 10 mM Ca^2+^ (open and closed circles, respectively). **A.** N153A. **B.** N153Q. **C.** D155A. **D.** D155E. **E.** D157A. **F.** D157E. **G.** D179A. **H.** D179E. The experimental data are the mean residue ellipticity at 222 nm ((Θ)_222_) monitored by far-UV CD.

**Figure 4:**
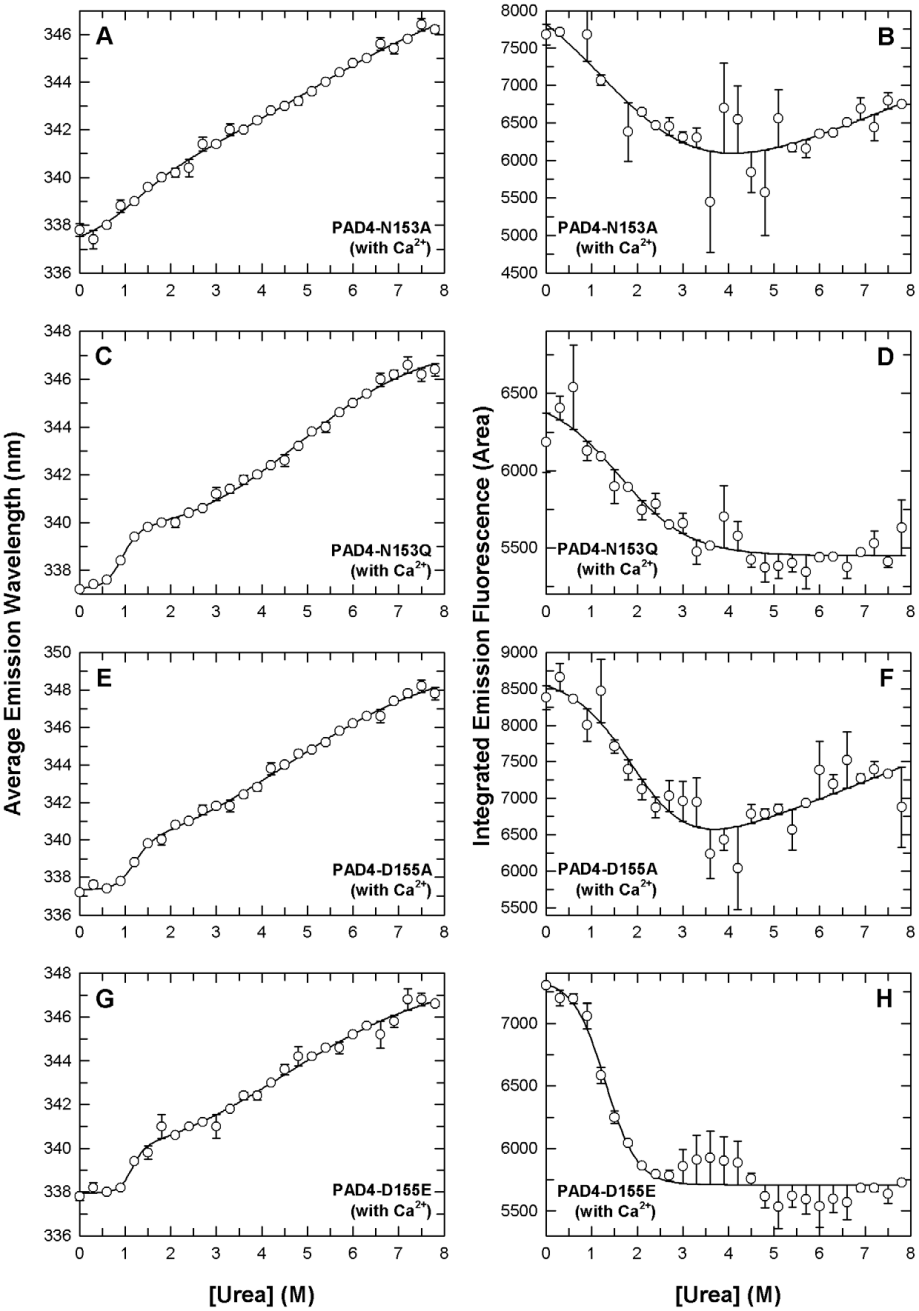
Denaturation curves of the PAD4 N153 and D155 mutant enzymes in the presence of calcium ions. PAD4 mutants in the presence of 10 mM Ca^2+^ were treated with various concentrations of urea in 30 mM Tris-HCl buffer (pH 7.4) at 25°C for 16 h and monitored using intrinsic protein fluorescence. Panels **A** (N153A), **C** (N153Q), **E** (D155A), and **G** (D155E) correspond to the experimental data of average emission wavelength, and panels **B** (N153A), **D** (N153Q), **F** (D155A), and **H** (D155E) correspond to the integrated emission fluorescence by intrinsic protein fluorescence. All data were fitted by either a two-state or three-state model. The fitting results and residues are shown as a solid line with error bars.

In urea-induced denaturation experiments of PAD4 mutants, the enzyme was preincubated with 10 mM Ca^2+^ to avoid protein precipitation during urea denaturation (Figure 4). For the N153A, N153Q, D155A and D155E enzymes, which displayed low catalytic power, the unfolding curves of the average fluorescence emission wavelength appeared to be biphasic, and a shoulder in the spectroscopic curves implied that an unstable intermediate existed at equilibrium (Figure 4, left panels). The [Urea]_0.5_ values of the N153A, N153Q, D155A and D155E mutants were less than WT, especially for the first phase. The [Urea]_0.5_ values were 1.1, 0.9, 1.2 and 1.2 M for the first phase (2.5 M for WT) and 5.2, 4.9, 4.6 and 4.5 M for the second phase (5.4 M for WT), respectively (Table 3). The denaturation curves of integrated emission fluorescence for the N153 and D155 mutants also behaved monophasically, which is similar to WT (Figure 4, right panels; however, they had smaller [Urea]_0.5_ values near 1.3-2.0 M (2.6 M for WT; Table 3), which indicated that these mutants were less stable than WT and more sensitive to urea denaturation even in the presence of Ca^2+^.

**Figure 5:**
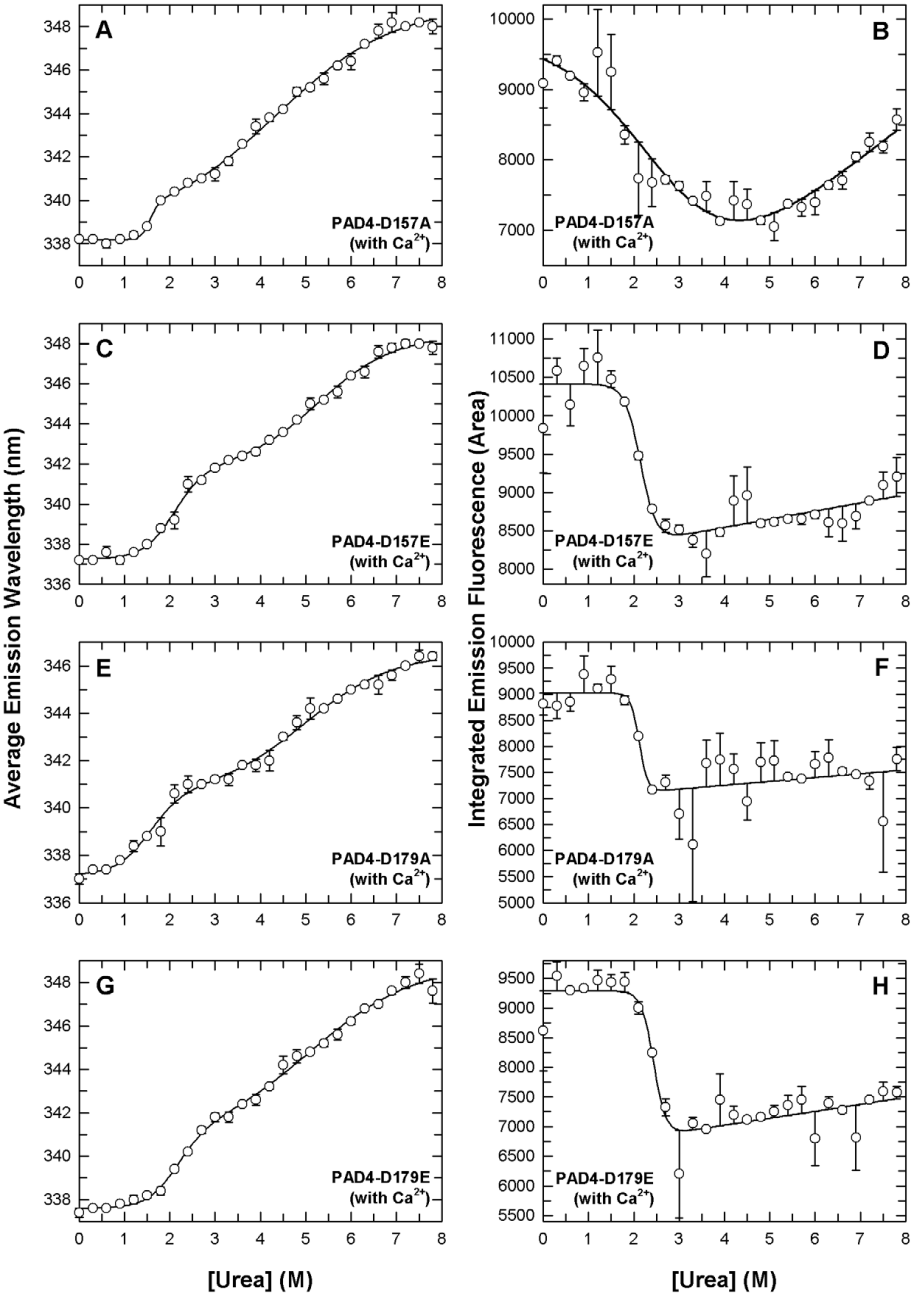
Denaturation curves of the PAD4 D157 and D179 mutant enzymes in the presence of calcium ions. PAD4 mutants in the presence of 10 mM Ca^2+^ were treated with various concentrations of urea in 30 mM Tris-HCl buffer (pH 7.4) at 25°C for 16 h and (D179E) correspond to the experimental data of average emission wavelength, and panels **B** monitored by intrinsic protein fluorescence. Panels **A** (D157A), **C** (D157E), **E** (D179A), and **G** (D157A), **D** (D157E), **F** (D179A), and **H** (D179E) correspond to the integrated emission fluorescence by intrinsic protein fluorescence. All data were fitted by either a two-state or three-state model. The fitting results and residues are shown as a solid line with error bars.

For the D157A and D179A enzymes, which also displayed low catalytic power, the average fluorescence emission wavelength curves were biphasic (Figure 5, left panels), and the [Urea]_0.5_ values were less than WT for the first phase. The [Urea]_0.5_ values of the D157A and D179A mutants were 1.6 M for the first phase (2.5 M for WT) and 4.3 and 5.0 M for the second phase (5.4 M for WT), respectively (Table 3). The denaturation curves of the integrated emission fluorescence for these two mutants were monophasic (Figure 5, right panels) with [Urea]_0.5_ values similar to WT (Table 3).

For the D157E and D179E enzymes, which displayed full catalytic power, the curves of the average fluorescence emission wavelength were biphasic (Figure 5, left panels) with [Urea]_0.5_ values of 2.1 and 2.2 M for the first phase (2.5 M for WT) and 5.3 and 5.1 M for the second phase (5.4 M for WT), respectively (Table 3). The unfolding curves of the integrated emission fluorescence for these two mutants were monophasic (Figure 5, right panels) with [Urea]_0.5_ values of 2.2 M and 2.4 M, respectively, which is similar to WT (2.6 M for WT; Table 3). These results demonstrate that most PAD4 mutants with low enzyme activity (N153A, N153Q, D155A, D155E and D157A) were less stable and more sensitive to urea than WT, which was indicated by the integrated emission fluorescence descending at the beginning of unfolding (Figure 4 and 5 , right panels), and their intermediate states appeared unstable (Figure 4 and 5, left panels). The thermal stabilities and unfolding patterns of D157E and D179E were more similar to that of WT, which suggests that the structural conformation of these two mutants is most similar to the WT in terms of enzyme catalysis and protein stability.

## Discussion

Ca^2+^ ions are essential for the enzymatic activity of PAD4, and replacement with other divalent ions does not activate PAD [39], [43], [44]. Structural evidence suggests that Ca^2+^ binding to the enzyme induces significant conformational changes that generate the active site cleft in the C-terminal domain. Binding of Ca^2+^ to the apoenzyme stabilizes disordered regions and organizes the active site [34]. In the crystal structures, Ca1 and Ca2 are located close to the binding site for the substrate complex; alanine substitution of one of the corresponding Ca^2+^-binding amino acids nearly completely abolished enzymatic activity. Therefore, these conformational changes are essential for catalysis, and Ca1 and Ca2 are defined as the catalytic Ca^2+^ ions.

In contrast, the exact role of the N-terminal Ca^2+^ ions, Ca3, Ca4 and Ca5, is not clear. Structural data revealed that Ca3, Ca4 and Ca5 stabilize a region from Asn158 to Val171 that is disordered in Ca^2+^-free PAD4 but is ordered in Ca^2+^-bound and substrate/complex-bound PAD4. In addition, sequence alignments of PAD isoforms indicate that the Ca^2+^-binding ligands in the active site and N-terminal site are well conserved. In this paper, we elucidate the significance of these N-terminal Ca^2+^ ions in enzyme catalysis and structural stability of PAD4.

### The Ca3 and Ca4 binding network is important for enzyme activation

Our kinetic studies clearly indicated that binding of Ca3 and Ca4 is essential for enzyme activation. Mutation of the amino acid residues Asp155, Asp157 and Asp179 that are coordinated with Ca3 and Ca4 (Figure 1B) caused the enzyme almost complete loss of catalytic power with large *K*_m,BAEE_ and *K*_0.5,Ca_ values, which indicates that the catalysis of the enzyme is significantly decreased by ablation of the negatively charged side chain of Asp at residue 155, 157 or 179 in the enzyme. This suggests that binding of Ca^2+^ ions at the Ca3 and Ca4 sites has a significant effect on the active site and that the Ca^2+^ ions in PAD4 can act as catalytic ions in the Ca1 and Ca2 binding sites and as stabilizing agents by binding to the N-terminal Ca^2+^-binding site. Like the active site cleft, the N-terminal Ca^2+^-binding site is also generated by binding calcium ions, which order the acidic residues in these regions to favor enzyme activation. Disruption of any one of the Ca3 and Ca4 binding ligands severely impaired the integration of the Ca3 and Ca4 binding network and resulted in an altered conformation in the N-terminal domain, which may have a significant influence on the Ca1 and Ca2 binding sites in the C-terminal domain, which prevents enzyme activation.

Three amino acid residues, Asn153, Asp165 and Asp176, are responsible for the binding network between Ca3 and Ca5 (Figure 1B). Mutation of Asn153, but not Asp165 or Asp176, to alanine resulted in a significant impact on the catalytic power of the enzyme, which suggests that the ligand interaction by Asn153 is the major force responsible for maintaining the exact structural geometry between Ca3 and Ca5. For Asn153 and Asp176, both residues display similar interactions with Ca3 and with Ca5 that are mediated by a water molecule, and they interact with each other. However, the N153A mutant was less active than the D176A mutant. This difference may be due to the additional hydrogen bonding between the amide side-chain of Asn153 and carboxylic side-chain of Glu252 that is indirectly coordinated with Ca5 by two water molecules. Abolishment of the side chain of Asn153 may disrupt the structural organization between Ca3 and Ca5 and severely interrupt the proper geometry required for enzyme activation.

### Ca^2+^ ions are essential for the conformational stability of PAD4

Our data suggests that the role of Ca^2+^ ions in PAD4 functions in catalysis and for global conformational stability of the enzyme. First, the *T*_m_ value of the Ca^2+^-bound enzyme is much higher than that of the Ca^2+^-free enzyme (Table 2). Second, the thermodynamic parameters derived from the urea-induced denaturation experiments of the N153, D155, D157 and D179 series mutants (Table 3) indicated that the binding of calcium ions in the N-terminal Ca^2+^-binding site is important for stabilizing the conformational geometry of the enzyme. Here, we provide direct evidence for the need of Ca3 and Ca4 in the N-terminal Ca^2+^-binding site, which is distal from the active site. The non-catalytic Ca^2+^ ions play a crucial role in the assembly of the correct geometry for full activation of the enzyme.

## Materials and Methods

### Expression and purification of recombinant peptidylarginine deiminase 4

Human PAD4 cDNA was cloned into the pQE30 vector, which contains an N-terminal His tag that was employed to purify the overexpressed PAD enzyme. This ampicillin-resistant vector was transformed into the JM109 strain of *E. coli*. The expression of PAD was induced with 1.0 mM isopropyl-1-thio-β-D-galactoside (IPTG), and the cells were harvested after incubation at 25°C overnight. Ni-NTA Sepharose (Sigma) was equilibrated with binding buffer (5 mM imidazole, 500 mM sodium chloride, 2.5 mM DTT, Triton X-100, 10% PMSF and 30 mM Tris-HCl, pH 7.6) for further purification. The lysate-Ni-NTA mixture was loaded onto a column and washed with a stepwise procedure (5, 10 and 20 mM imidazole washes in buffer containing 500 mM sodium chloride, 2.5 mM DTT and 30 mM Tris-HCl, pH 7.6) to minimize the unwanted proteins. Finally, PAD enzymes were eluted with elution buffer (80 mM imidazole, 500 mM sodium chloride, 2.5 mM DTT and 30 mM Tris-HCl, pH 7.6). The purity of the enzymes was examined by SDS-PAGE, and the protein concentrations were determined using the Bradford method.

### Site-directed mutagenesis

Site-directed mutagenesis was performed using a QuikChange™ kit (Stratagene, La Jolla). This mutagenesis method was performed using *Pfu* DNA polymerase, which replicates both plasmid strands with high fidelity in 16- to 20-cycle PCR runs. Mutagenic primers were 25- to 45-mer oligonucleotides that specifically bound the template DNA. Mutations of the Ca^2+^ binding site residues were selected. The synthetic oligonucleotides used as mutagenic primers were as follows:

N153A 5′-GCCATCCTGCTGGTGGCTTGTGACAGAGACAATC-3′

N153D 5′-GCCATCCTGCTGGTGGACTGTGACAGAGACAATC-3′

N153Q 5′-GCCATCCTGCTGGTGCAGTGTGACAGAGACAATC-3′

D155A 5′-CCTGCTGGTGAACTGTGCTAGAGACAATCTCG-3′

D155E 5′-CCTGCTGGTGAACTGTGAAAGAGACAATCTCG-3′

D155N 5′-CCTGCTGGTGAACTGTAACAGAGACAATCTCG-3′

D157A 5′-GGTGAACTGTGACAGAGCTAATCTCGAATCTTCTG-3′

D157E 5′-GGTGAACTGTGACAGAGAAAATCTCGAATCTTCTG-3′

D157N 5′-GGTGAACTGTGACAGAAACAATCTCGAATCTTCTG-3′

D165A 5′-GAATCTTCTGCCATGGCTTGCGAGGATGATG-3′

D168A 5′-GCCATGGACTGCGAGGCTGATGAAGTGCTTGAC-3′

D176A 5′-GTGCTTGACAGCGAAGCTCTGCAGGACATGTCG-3′

D179A 5′-GCGAAGACCTGCAGGCTATGTCGCTGATGAC-3′

D179E 5′-GCGAAGACCTGCAGGAAATGTCGCTGATGAC-3′

D179N 5′-GCGAAGACCTGCAGAACATGTCGCTGATGAC-3′

E252A 5′-CATGGACTTCTACGTGGCTGCCCTCGCTTTCCCG-3′

D388A 5′-GAGTGATGGGTCCAGCTTTTGGCTATGTAAC-3′.

### Enzyme assay and kinetic data analysis

The protocol for the continuous assay of PAD4 activity was previously reported [39]. The standard reaction mixture for spectrophotometric assay of PAD4 contains 10 mM benzoyl-L-arginine ethyl ester (BAEE) as an artificial substrate, 10 mM CaCl_2_, 2.5 mM dithiothreitol (DTT), 8.5 mM α-ketoglutarate (α-KG), 0.22 mM NADH and 6 U of glutamate dehydrogenase (GDH) in 100 mM Tris-HCl (pH 7.5) in a 1-ml cuvette at 37°C. An appropriate amount of PAD4 is added to the assay mixture to initiate the reaction. After adding enzyme to the reaction, the decrease in absorbance at 340 nm was continuously traced using a Perkin-Elmer Lamba-25 spectrophotometer. An enzyme unit is defined as the amount of enzyme that catalyzes the production of 1 µmol of NADH per min. An absorption coefficient of 6220 cm^−1^M^−1^ at 340 nm for NADH was used in calculations. The apparent Michaelis constant for the BAEE substrate was determined by varying the BAEE concentration near its *K*_m_ value, with the concentration of other components maintained. The experimental data were analyzed using Prism 4.0.

The sigmoidal curves of [Ca^2+^] versus initial rates were fitted to the Hill equation, and the data were further analyzed to calculate the *K*_0.5,Ca_ value (i.e., the calcium concentration at half-maximal velocity) and the Hill coefficient (*h*), which were employed to assess the degree of cooperativity.
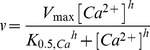



All data fitting were performed using Sigma Plot 8.0 (Jandel, San Rafael, CA).

### Thermal stability experiments by circular dichroism spectrometry

The thermal denaturation of PAD4 and mutant enzymes was performed in 30 mM Tris-HCl (pH 7.4). Circular dichroism (CD) spectrometry was performed using a Jasco J-815 spectropolarimeter with 0.1-cm quartz cuvettes and a 1-nm slit bandwidth. The ellipticity at 222 nm of all samples was recorded to analyze the protein conformational changes that occurred during the thermal denaturation process. The mean residue ellipticity ((Θ)) at 222 nm was calculated using the following equation:
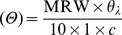
where MRW is the mean residue weight, θ_λ_ is the measured ellipticity in degrees at wavelength λ, l is the cuvette pathlength (0.1 cm), and c is the protein concentration in g/ml.

### Conformational stability experiments by fluorescence

The urea-induced denaturation of PAD4 and mutant enzymes was performed in the presence of 10 mM CaCl_2_ in 30 mM Tris-HCl buffer (pH 7.4) at 25°C for 16 h. Fluorescence spectra of the proteins were monitored using a Hitachi F-4500 FL luminescence spectrometer at 25°C, and all spectra were corrected for buffer absorption. The Raman spectrum of water was corrected. The excitation wavelength was set to 290 nm, and the fluorescence emission spectra were scanned from 300 to 400 nm. The average emission wavelength (<*λ*>) was analyzed using the average emission wavelength method [40] and calculated according to the following equation:
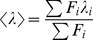
in which *F_i_* is the fluorescence intensity at the specific emission wavelength (*λ_i_*).

### Analysis of the urea-induced denaturation curve

Analysis of the unfolding curves of the denaturation process was performed as described by Pace [41] and assumed a two-state or three-state unfolding mechanism. For a two-state model, Δ*G*_(H2O)_ and *m* values were estimated by fitting the data to the following equation:
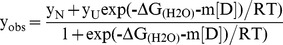
where *y*_obs_ denotes the observed signal change, and *y*_N_ and *y*_U_ represent the signals of the folded and unfolded states, respectively. Δ*G*_(H2O)_ denotes the intrinsic free energy change in the absence of denaturant, and *m* represents the dependence of Δ*G* on the denaturant. [D] denotes the denaturant concentration, *T* is the absolute temperature in degrees Kelvin, and *R* is the gas constant.

The denaturation curve was analyzed using a three-state model. Δ*G*_(H2O)_ and *m* values at each step were estimated by fitting the overall data to the following equation:
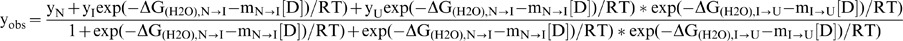



where *y*_I_ represent the signals of the intermediate states, Δ*G*_(H2O),N→I_ and Δ*G*_(H2O),I→U_ denote the intrinsic free energy change for the native to intermediate (N→I) and for the intermediate to denatured (I→U) processes, respectively, and *m*_ N→I_ and *m*_ I→U_ are the *m* values for the corresponding processes. The concentration of urea for half-denaturation of the protein, [Urea]_0.5_ was estimated by dividing Δ*G* by *m* [41].

### Quaternary structure analysis by analytical ultracentrifugation

Sedimentation velocity experiments were performed using a Beckman Optima XL-A analytical ultracentrifuge. Sample (380 µl) and buffer (400 µl) solutions were separately loaded into the double sector centerpiece and placed in a Beckman An-50 Ti rotor. Experiments were performed at 20°C and at a rotor speed of 42,000 rpm. Protein samples were monitored by UV absorbance at 280 nm in a continuous mode with a time interval of 480 s and a step size of 0.002 cm. Multiple scans at different time points were fitted to a continuous size distribution model by the program SEDFIT [42]. All size distributions were solved at a confidence level of p = 0.95, a best fitted average anhydrous frictional ratio (*f/f_0_*), and a resolution N of 200 sedimentation coefficients between 0.1 and 20.0 S.

## Supporting Information

Figure S1 Continuous sedimentation coefficient distributions of WT PAD4 and the mutants. The enzymes (0.3 mg/ml) were in 30 mM Tris-acetate (pH 7.4) at 25°C for 16 h and then run in an analytical ultracentrifuge at 20°C. **A.** WT. **B.** N153A. **C.** D155A. **D.** D157A. **E.** D179A.
(TIF)
