# Supplementary figures and images for: Functional Roles of the Non-Catalytic Calcium-Binding Sites in the N-Terminal Domain of Human Peptidylarginine Deiminase 4
Source: PLoS One. 2013 Jan 30;8(1):e51660. doi: 10.1371/journal.pone.0051660 (PMC3559651; doi:10.1371/journal.pone.0051660)

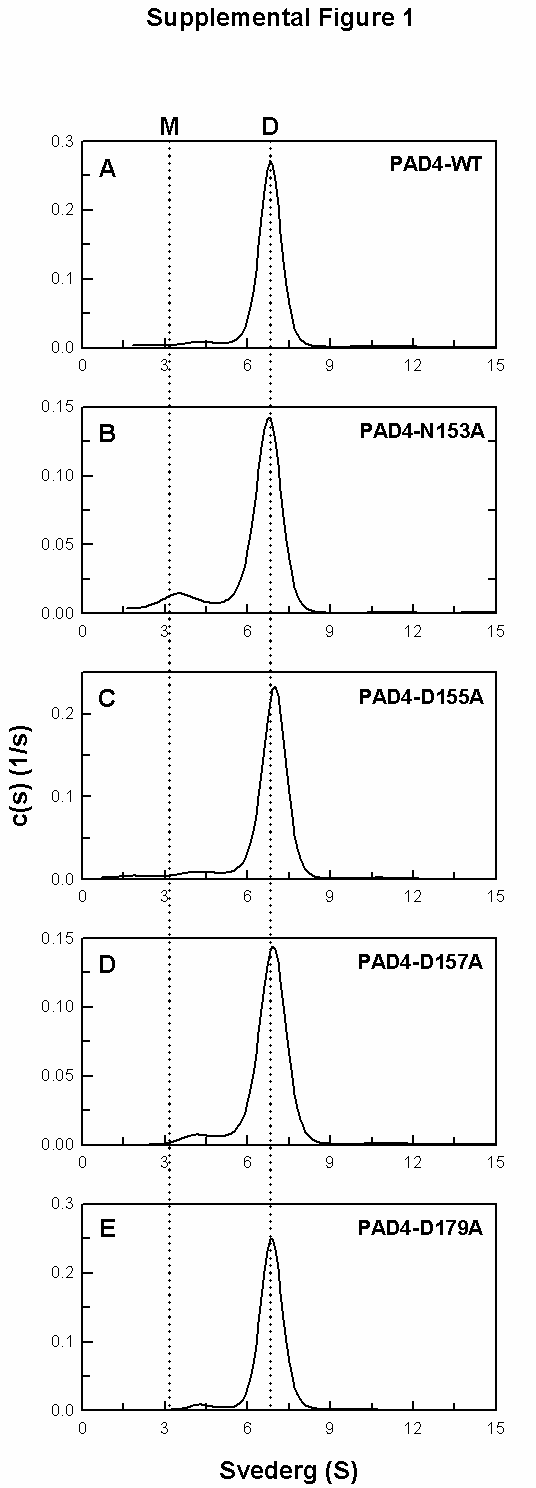

Supplement: Figure S1 — Continuous sedimentation coefficient distributions of WT PAD4 and the mutants. The enzymes (0.3 mg/ml) were in 30 mM Tris-acetate (pH 7.4) at 25°C for 16 h and then run in an analytical ultracentrifuge at 20°C. A. WT. B. N153A. C. D155A. D. D157A. E. D179A. (TIF) [file pone.0051660.s001.tif]
